# Exploring the Pharmacological Potential of Carrageenan Disaccharides as Antitumor Agents: An In Silico Approach

**DOI:** 10.3390/md23010006

**Published:** 2024-12-26

**Authors:** Ohana Leticia Tavares Silva, Monique Gabriela das Chagas Faustino Alves, Hugo Alexandre Oliveira Rocha

**Affiliations:** Graduate Program in Biochemistry and Molecular Biology, Center of Biosciences, Federal University of Rio Grande do Norte—UFRN, Av. Sen. Salgado Filho, 3000, Natal 59078-900, Brazil; ohana.tavares.705@ufrn.edu.br (O.L.T.S.); monique.alves@ufrn.br (M.G.d.C.F.A.)

**Keywords:** sulfated polysaccharides, bioinformatics, red seaweed, cancer

## Abstract

Carrageenans have demonstrated enhanced antitumor activity upon depolymerization into disaccharides. However, the pharmacological viability of these disaccharides and their mechanisms of antitumor action remains to be fully elucidated. This study aimed to employ computational tools to investigate the pharmacological properties and molecular targets pertinent to cancer of the disaccharides derived from the primary carrageenans. Analyses of pharmacological properties predicted by the pkCSM and SwissADME servers indicated that the disaccharides possess a favorable pharmacokinetic profile, although they encounter permeability challenges primarily due to their high polarity and low lipophilicity. Target prediction using SwissTarget and PPB2 identified five carbonic anhydrases, which are also targets of oncology drugs, as common targets for the disaccharides. Molecular docking performed with AutoDock Vina revealed that the binding energies of the disaccharides with carbonic anhydrases were comparable to or greater than those of existing drugs that target these lyases. Notably, six of the complexes formed exhibited interactions between the disaccharides and the zinc cofactor, which represents a primary mechanism of inhibition for these targets. Furthermore, molecular dynamics simulations conducted using GROMACS demonstrated a stable interaction between the disaccharides and carbonic anhydrases. These findings offer new insights into the pharmacological properties and mechanisms of action of carrageenan-derived disaccharides, highlighting their potential for further exploration in clinical trials and experimental studies.

## 1. Introduction

According to the World Health Organization (WHO), cancer is the second leading cause of death worldwide, accounting for nearly 10 million deaths in 2020 [1]. This underscores the urgent need for new strategic plans for its control. Cancer refers to a group of more than 100 diseases, characterized by the uncontrolled growth of cells that can spread throughout the body, driven by the accumulation of genetic mutations [2,3]. These genetic alterations confer a set of common phenotypic characteristics on cancer cells, including the ability to evade growth suppressors, escape immune destruction, achieve immortality, promote tumor-associated inflammation, activate invasion and metastasis, induce or access vasculature, exhibit genomic instability and mutations, resist cell death, deregulate metabolism, and sustain proliferative signaling [4]. These traits are essential for the survival of cancer cells and, as such, are the focus of intense scientific investigation, particularly for the development of novel therapies [5].

Currently, the most frequently used therapies for cancer treatment include surgery, chemotherapy, radiotherapy, targeted therapy, immunotherapy, and hormone therapy. However, while radiotherapy and chemotherapy are effective, they are highly toxic to non-tumor cells due to their low selectivity. This lack of specificity can lead to significant adverse effects, such as pain, nausea, diarrhea, cardiotoxicity, hair loss, skin problems, and immune system suppression [6]. More selective treatments, such as targeted therapy, immunotherapy, and hormone therapy, though causing fewer adverse effects on normal cells, also face limitations due to the heterogeneity of tumors in terms of therapeutic targets, as well as the development of mutations that reduce the effectiveness of these treatments [7]. Considering these challenges, natural products have garnered attention as potential sources of new therapeutic agents, due to their potential for higher selectivity toward tumor cells, thus reducing the incidence of adverse effects [8]. Studies indicate that nearly 60% of drugs approved for cancer treatment have their origins in natural compounds [9]. Among emerging natural sources, seaweeds have attracted significant interest from pharmaceutical companies and academic institutions in marine biotechnology, as they offer substantial potential for the discovery of novel anticancer compounds, such as sulfated polysaccharides (SP) [10,11,12,13,14,15].

Sulfated polysaccharides are found across the three major taxa of macroalgae: brown algae (Phaeophyta), red algae (Rhodophyta), and green algae (Chlorophyta). The primary SP in red algae are galactans, commercially recognized as agarans and carrageenans, whereas the predominant SP in brown algae are fucans (homofucans) and fucoidans (heterofucans). In contrast, the principal SP in green algae are sulfated mono- and heteropolysaccharides, which include sugars such as galactose, xylose, arabinose, mannose, glucuronic acid, and/or glucose [16].

Carrageenans, extracted from red seaweed, are structurally composed of alternating units of D-galactose and 3,6-anhydro-galactose, linked by α-1,3 and β-1,4 glycosidic bonds [17]. There are at least 15 types of carrageenans, categorized based on their structural characteristics, including sulfation patterns and the presence or absence of anhydro-galactose (AnGal). The primary types are kappa (κ), iota (ι), and lambda (λ) carrageenans [16].

Although carrageenans are widely utilized as stabilizers and thickeners in the textile, food, cosmetic, and pharmaceutical industries [18], both in vitro and in vivo studies have demonstrated that these polysaccharides are effective in inhibiting various tumor development processes [19,20]. However, their high molecular mass (MM) and complex structures pose significant challenges for clinical development and therapeutic application [21]. In this context, oligosaccharides, including disaccharides derived from these polysaccharides, represent a promising alternative due to their smaller size and less complex structures [21,22].

Previous studies have demonstrated that the depolymerization of carrageenans, particularly κ-, ι-, and λ-carrageenan, enhances their antitumor activity [23,24]. However, it remains unclear whether these disaccharides/oligosaccharides are pharmacologically viable, and the precise mechanisms by which they inhibit tumor growth are still unknown.

Computational tools have proven invaluable in the investigation of compounds and drugs, facilitating the prediction of pharmacokinetic properties and the safety profiles of lead candidates—compounds with pharmacological potential—through comprehensive analysis of ADMET (Absorption, Distribution, Metabolism, Excretion, and Toxicity) profiles [25]. Furthermore, these tools enable the identification of physicochemical characteristics that align with those of approved drugs via drug-likeness testing [26]. The exploration of potential pharmacological targets for specific compounds is achievable through techniques such as target prediction based on similarity to known ligands, docking studies, and molecular dynamics simulations [27]. Collectively, these methodologies enhance the accuracy and efficiency of new compound discovery, while significantly reducing the time and costs associated with experimental procedures [28]. Moreover, they provide critical insights into the potential efficacy of carrageenans and their derived compounds.

Considering the aforementioned considerations, the present study aimed to investigate the most representative disaccharide units of the three predominant types of carrageenan—iota, kappa, and lambda—utilizing computational tools to elucidate aspects of pharmacokinetics, potential toxicity, physicochemical similarity with established drugs, and possible targets associated with their antitumor activity.

This study is justified by the urgent need for innovative cancer treatments, the promising therapeutic applications of sulfated polysaccharides derived from seaweed, and the implementation of computational methodologies that can streamline the identification of new anticancer agents. The exploration of disaccharide units from the three primary types of carrageenan will not only contribute to the advancement of scientific knowledge but may also unveil new therapeutic opportunities in the ongoing fight against cancer.

## 2. Results

### 2.1. ADMET Profile

#### 2.1.1. Absorption

When evaluated for absorption (Table 1), the disaccharides demonstrated a log (logarithm of water solubility) that characterizes them as water soluble [29]. However, none of the compounds exhibited high permeability in Caco-2 cells (<0.90), an in vitro model commonly used to predict intestinal absorption. The iota (dIC) and lambda (dLC) carrageenan disaccharides showed no detectable evidence of intestinal (human) absorption, while the kappa (dKC) carrageenan disaccharide exhibited an absorption rate below the 30% threshold. Both displayed skin permeability < −2.5. Furthermore, they were identified as P-glycoprotein (P-gp) substrates but not as inhibitors of its isoforms I and II.

#### 2.1.2. Distribution

In the distribution tests (Table 1), all disaccharides exhibited VDss (Volume of Distribution at steady state) values below −0.15, indicating low distribution throughout the body. Regarding permeability to the blood–brain barrier (BBB) and central nervous system (CNS), dIC, dKC, and dLC displayed values below the thresholds of −1 for BBB penetration and −3 for CNS permeability, suggesting limited access to these regions.

#### 2.1.3. Metabolism and Excretion

Regarding metabolism (Table 1), dIC, dKC, and dLC were not identified as substrates of the Cytochrome P450 (CYP) isoforms CYP2D6 and CYP3A4. Additionally, none of the disaccharides exhibited inhibitory activity against the CYP isoforms CYP1A2, CYP2C19, CYP2C9, CYP2D6, or CYP3A4. In terms of excretion, dKC displayed the lowest log total clearance, followed by dIC and dLC. None of the compounds were identified as substrates of the renal transporter OCT2 (Table 1).

#### 2.1.4. Toxicity

In toxicity tests (Table 2), the disaccharides did not demonstrate the ability to cause mutations in bacteria (AMES). The maximum recommended tolerated dose (MRTD) for use in humans was greater than 0.477 for dIC and dKC and equal to 0.477 for dLC. The results of the hERG I and hERG II inhibition test indicated that these disaccharides do not inhibit these potassium channels. In acute oral toxicity in rats, the LD50 (amount of a substance required to kill 50% of the animals in a group) of dKC was the lowest, and that of dLC was the highest. Regarding chronic oral toxicity in rats, the LOAEL (lowest dose at which the adverse effect is observed) values of dIC were the lowest found, followed by dKC and dLC. None of the molecules were shown to cause hepatotoxicity or skin sensitivity. Flathead Minnow fish were also not toxic, with LC50 (concentration of a molecule required to cause the death of 50% of the animals) greater than 0.5. However, in the toxicity test on *Tetrahymena pyriformis*, the plGC50 values (negative logarithm of the concentration required to inhibit 50% of growth) were greater than the limit of −0.5.

### 2.2. Drug-Likeness Physicochemical Property Analysis

In the drug-likeness evaluations (Table 3), the Lipinski test revealed that the dIC and dKC disaccharides violated only one criterion, with H-acceptors (hydrogen acceptors) exceeding 10. Conversely, the dLC disaccharide violated two criteria: MW (molecular weight) > 500 g/mol and H-acceptors > 10. In the Ghose test, all disaccharides exhibited a WLOGP (Wildman and Crippen logarithm of the octanol-water partition coefficient) value below −0.4, and the MW of dLC exceeded the 480 g/mol threshold. In both the Veber and Egan tests, the only common violation across all disaccharides was a TPSA (topological polar surface area) than the threshold value proposed by the tests. In the Muegge test, which assesses a broader set of parameters, all disaccharides presented three violations: XLOGP3 (octanol–water partition coefficient) < −2, TPSA > 150 Å^2^, and H-acceptors > 10.

### 2.3. Identification of Cancer-Related Molecular Targets

The results showed significant convergence in the identification of potential targets for the carrageenan disaccharides. Initially, the SwissTargetPrediction and PPB2 tools returned 15 and 20 targets, respectively, for dIC, dKC, and dLC. Among these, 14 targets predicted by SwissTargetPrediction were common to all three disaccharides (Table S1). In PPB2, 18 targets were shared between dIC, dKC, and dLC (Table S2). By cross-referencing the common targets from both tools, 5 overlapping targets were identified: Carbonic Anhydrases (CA) I, II, IX, XII, and XIV. These targets were ranked between 1st and 5th in terms of the likelihood of bioactivity for each disaccharide (Table 4).

The dataset downloaded from Probes and Drugs, which contains approximately 163 drugs approved for cancer treatment by the NIH (National Institutes of Health), along with their respective targets, enabled further analysis of the identified disaccharide targets (Table S3). It was found that all final targets for the disaccharides are also targeted by existing oncology drugs (Table 4). For example, celecoxib, imatinib, nilotinib, and bortezomib are known to target CA I, CA II, CA IX, CA XII, and CA XIV. Additionally, zoledronic acid targets CA II, CA IX, CA XII, and CA XIV, hydroxyurea targets CA II and CA IX, and pazopanib targets CA IX.

### 2.4. Molecular Docking

In the docking studies between the disaccharides and the final targets, dIC exhibited binding energies of −9.7, −9.1, −8.0, −9.3, and −8.4 kcal/mol; dKC showed scores of −9.2, −8.3, −8.6, −8.8, and −8.5 kcal/mol; and dLC achieved values of −10.3, −9.2, −8.6, −9.8, and −10.8 kcal/mol for the enzymes CA I, CA II, CA IX, CA XII, and CA XIV, respectively (Table 5). When compared with the docking scores of oncology drugs already approved for these targets (Table S4), dIC and dLC surpassed the binding energy of all drugs targeting CA I, while dKC outperformed three drugs (celecoxib, imatinib, and bortezomib). In dockings with CA II, dIC and dLC showed better binding energies than three of the six drugs (hydroxyurea, bortezomib, and zoledronic acid), while dKC outperformed hydroxyurea and zoledronic acid. For CA IX, hydroxyurea and zoledronic acid had lower binding energies than all three disaccharides, and dKC and dLC also surpassed the binding energy of bortezomib. In the case of CA XII, dIC and dLC outperformed all drugs for this target except for nilotinib, which had a higher binding energy, while dKC fell behind both nilotinib and celecoxib. Finally, in the interactions with CA XIV, dIC and dKC only surpassed zoledronic acid, while dLC exhibited superior binding energies compared to all drugs acting on this target.

**Figure 1:**
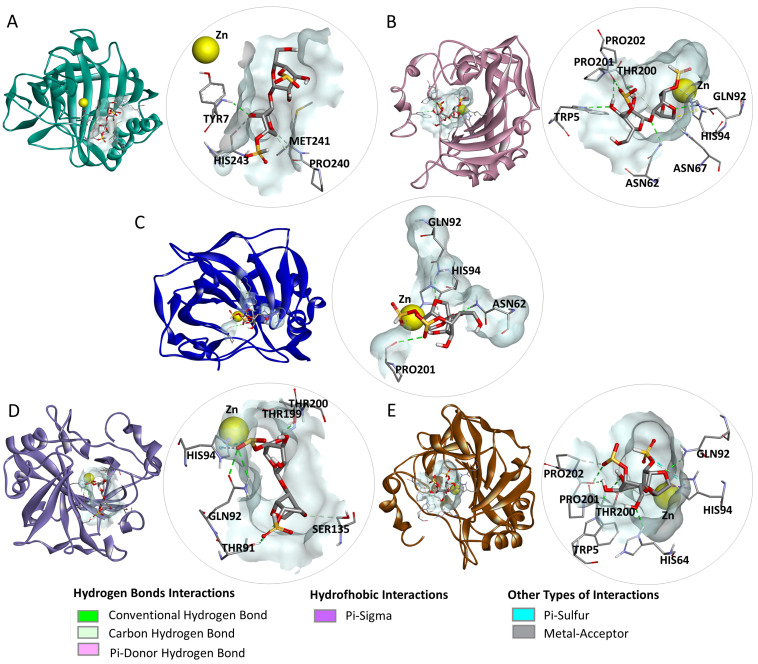
Interactions performed in the docking of the iota carrageenan disaccharide (dIC) with carbonic anhydrase (CA) I, II, IX, XII, and XIV: (**A**) dIC-CAl complex, (**B**) dIC-CAII complex, (**C**) dIC-CAIX complex, (**D**) dIC-CAXII complex, and (**E**) dIC-CAXIV complex.

In the analysis of the interactions of the complexes (Figure 1), it was observed that dIC formed hydrogen bonds with the amino acid residues TYR7, MET241, PRO240, and HIS243 of CA I; TRP5, ASN62, GLN92, THR200, PRO201, HIS94, PRO202, and ASN67 of CA II; ASN62, GLN92, PRO201, and HIS94 of CA IX; THR91, GLN92, THR199, THR200, SER135, and HIS94 of CA XII; and TRP5, HIS64, GLN92, THR200, PRO201, and PRO202 of CA XIV. In addition, hydrophobic interactions were observed with HIS94 in CA II and CA XIV, alongside other interactions involving the zinc (Zn) cofactor in CA IX, and with HIS94 in CA XII and CA XIV.

**Figure 2:**
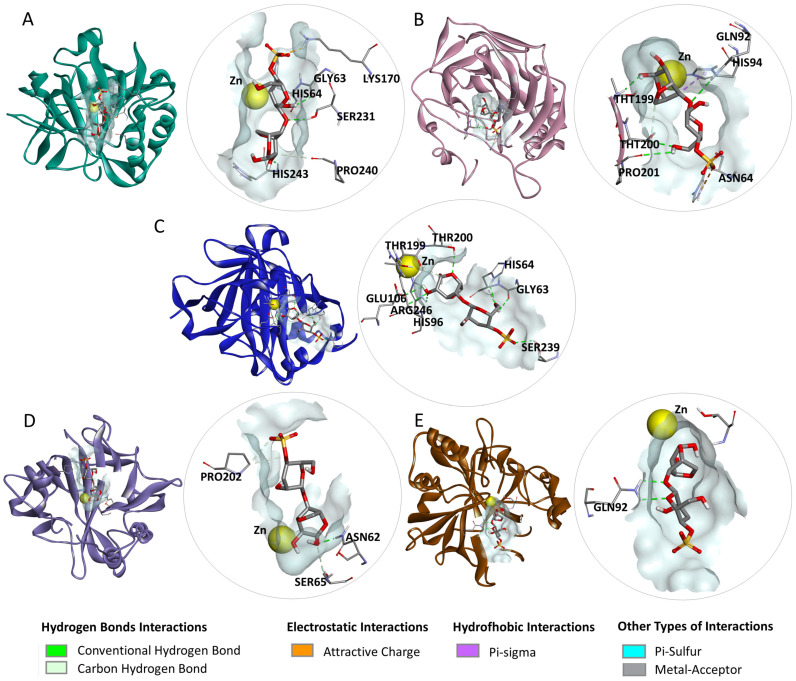
Interactions observed in the docking studies of the disaccharide kappa carrageenan (dKC) with carbonic anhydrases (CAs) I, II, IX, XII, and XIV: (**A**) dKC-CA I complex, (**B**) dKC-CA II complex, (**C**) dKC-CA IX complex, (**D**) dKC-CA XII complex, and (**E**) dKC-CA XIV complex.

The dKC (Figure 2) exhibited hydrogen bond interactions with residues LYS170, SER231, HIS243, GLY63, HIS64, and PRO240 of carbonic anhydrase I (CA I); GLN92, THR199, PRO201, and THR200 of carbonic anhydrase II (CA II); HIS64, HIS96, THR200, SER239, ARG246, GLU106, GLY63, and THR199 of carbonic anhydrase IX (CA IX); ASN62, SER65, and PRO202 of carbonic anhydrase XII (CA XII); as well as GLN92 of carbonic anhydrase XIV (CA XIV). Additionally, it demonstrated electrostatic interactions with LYS170 of CA I and HIS64 of CA II, hydrophobic interactions with HIS94 of CA II, and an interaction with the cofactor (Zn) of the enzyme CA II.

**Figure 3:**
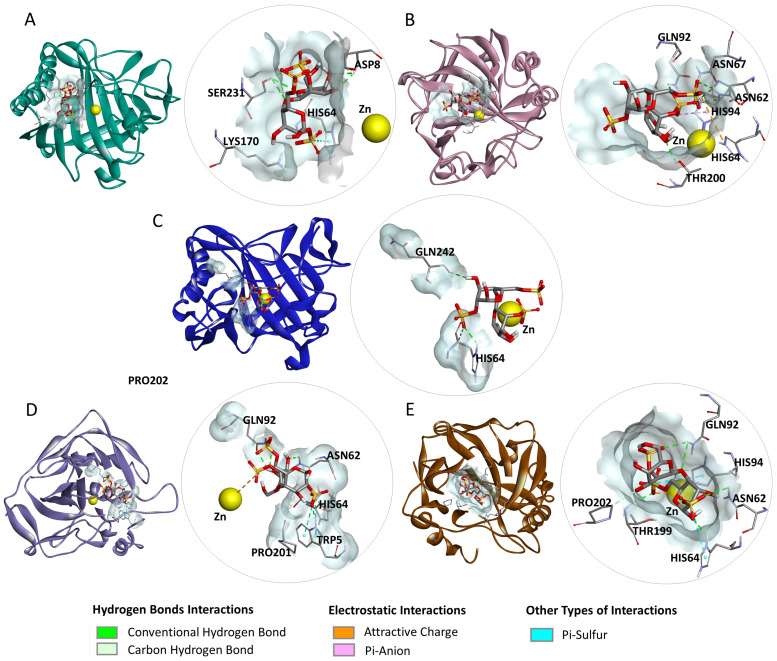
Interactions performed in the dockings of the disaccharide lambda carrageenan (dLC) with carbonic anhydrase (CA) I, II, IX, XII, and XIV: (**A**) dLC-CAI complex, (**B**) dLC-CAII complex, (**C**) dLC-CAIX complex, (**D**) dLC-CAXII complex, and (**E**) dLC-CAXIV complex.

In the interactions of dLC (Figure 3), the amino acid residues that formed hydrogen bonds included ASP8 and SER231 of carbonic anhydrase I (CA I); ASN62, ASN67, GLN92, and THR200 of carbonic anhydrase II (CA II); HIS64 and GLN242 of carbonic anhydrase IX (CA IX); TRP5, ASN62, GLN92, and PRO201 of carbonic anhydrase XII (CA XII); as well as ASN62, HIS64, GLN92, THR199, HIS94, and PRO202 of carbonic anhydrase XIV (CA XIV). Additionally, electrostatic interactions were established by residues LYS170 of CA I, HIS64 of CA II, CA XII, and CA XIV, HIS94 of CA II, as well as the Zn ion of CA XII and CA XIV. Furthermore, HIS64 of CA I, TRP5 of CA XII, and HIS64 and HIS94 of CA XIV participated in other types of interactions with dLC.

### 2.5. Molecular Dynamics Simulations

**Figure 4:**
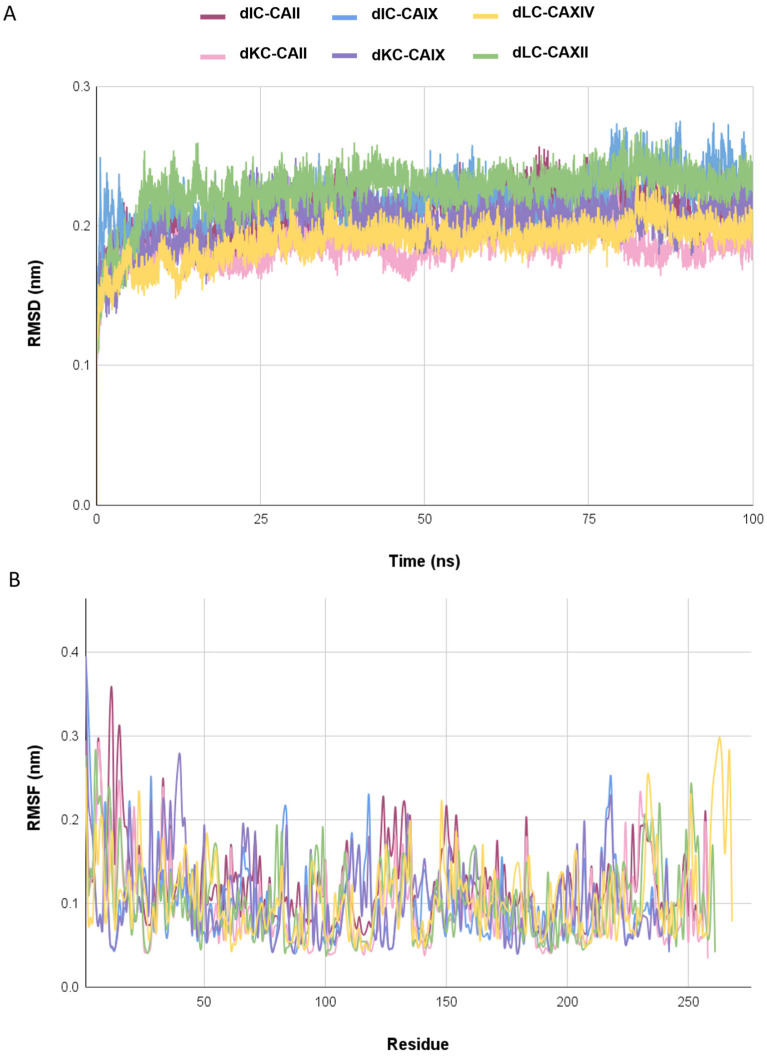
Plots extracted from molecular dynamics simulations of the complexes dIC-CAII, dKC-CAII, dIC-CAIX, dKC-CAIX, dLC-CAXII, and dLC-CAXIV for 100 ns: (**A**) Root mean square deviation (RMSD), (**B**) Root mean square fluctuation (RMSF).

The RMSD (root mean square deviation) plot indicates that all complexes achieved a stable state, with RMSD values consistently below 0.28 nm throughout the equilibrium phase (Figure 4A). The dKC-CA II and dLC-CA XIV complexes exhibited an average RMSD value of 0.19 nm, while the dIC-CA II and dKC-CA IX complexes had an average of 0.21 nm. The dIC-CA IX and dLC-CA XII complexes demonstrated average RMSD values of 0.22 nm and 0.23 nm, respectively.

In the RMSF (root mean square fluctuation) plot (Figure 4B), greater fluctuations were observed in the residues located at the terminal regions of the enzymes, with RMSF values exceeding 0.3 nm in the dIC-CA IX and dIC-CA II complexes. Conversely, regions near the binding sites, particularly the residues directly involved in ligand interactions, exhibited smaller fluctuations, ranging from 0.1 to 0.2 nm across all complexes. Regarding the average RMSF values, the dKC-CA II, dIC-CA IX, dLC-CA XII, and dLC-CA XIV complexes displayed values of 0.10 nm, while the dKC-CA IX complex recorded 0.11 nm, and the dIC-CA II complex registered 0.12 nm.

## 3. Discussion

The in silico evaluation of the ADMET properties of a molecule has increasingly become a crucial component in the investigation of new drug candidates. This approach provides valuable information more rapidly and economically regarding their potential pharmaceutical and toxicological outcomes [30,31].

Understanding the ADMET characteristics of a substance in the human body is essential for predicting the pharmacokinetic and pharmacodynamic profiles of a molecule, regardless of whether it is classified as a drug [32]. Currently, a variety of systems—both offline and online software—are available to assist researchers in anticipating the behavior of a molecule within the human body.

In the results of the ADMET profile of carrageenan disaccharides, high solubility was observed, which is a highly sought-after characteristic in drugs, since solubility helps in the processes of absorption, distribution, metabolism, and excretion of the molecule in the body [33].

However, the permeability of the disaccharides to Caco-2 cells and the intestinal barrier was found to be unsatisfactory. This observation can be attributed to the recognition of these disaccharides as substrates for P-gp, which functions as an efflux pump in biological membranes. P-gp actively transports its substrates back into the lumen, thereby reducing the permeability of compounds [34]. Conversely, carrageenan disaccharides do not inhibit this protein, which may help prevent the unwanted accumulation of other substances in tissues and mitigate the risk of undesirable drug interactions [35].

Another factor that influences the permeability of Caco-2 cells and the intestinal barrier is the presence of sulfate groups in the disaccharides. These sulfate groups impart a negative charge to the disaccharides, leading to repulsion with the negatively charged molecules of the glycocalyx, particularly glycosaminoglycans [36].

Despite these data, this characteristic alone should not preclude the consideration of disaccharides as potential antitumor agents. Notably, several FDA-approved antitumor drugs, such as paclitaxel, etoposide, gemcitabine, docetaxel, and vinorelbine, also exhibit low absorption values in the gastrointestinal tract. Additionally, some of these drugs have reduced bioavailability due to the action of the P-gp [37].

Regarding absorption, the data indicate that disaccharides exhibit skin permeability, suggesting a potential avenue for the administration of these compounds, particularly in the context of treating skin cancers. This possibility is supported by findings that the α-carrageenan disaccharide demonstrates cytotoxic effects against murine melanoma cells (B16-F10), with an IC50 of 0.039 mg/mL [22].

In the context of distribution, low values of VDss indicate a slower distribution of these disaccharides within tissues. Consequently, it may be necessary to administer these compounds more frequently and in smaller doses to sustain adequate therapeutic levels [38]. Furthermore, these compounds exhibit low permeability across the BBB and into the CNS, a limitation that is influenced by recognition by P-gp, thereby restricting their activity in this region [34].

Specifically in relation to the disaccharides studied here, there are two factors in their pharmacokinetics that act positively on their antitumor action: their metabolism and excretion.

Regarding metabolism, disaccharide molecules are not recognized as substrates of CYP enzymes and are therefore not metabolized by them. This characteristic may be advantageous, as CYP-mediated metabolism can eliminate a significant proportion of certain orally administered drugs before they reach systemic circulation [39]. Furthermore, metabolism by CYP enzymes is often associated with tumor cell resistance to drugs, particularly those used in cancer therapy [40].

Another positive aspect is that these disaccharides do not inhibit the isoforms of CYP enzymes, indicating that they do not interfere with CYP-mediated metabolism and are unlikely to alter the metabolism of other substances, including drugs and toxins [41].

Excretion studies suggest that disaccharides are not actively excreted by the renal transporter OCT2 and exhibit a slower total clearance profile. This property contributes to their prolonged presence in the body and enhances their bioavailability [42].

In toxicity assessments, disaccharides have not been identified as mutagenic agents and are not associated with an increased risk of cancer in the Ames test [43]. The MRTD values observed for these disaccharides were notably high, suggesting that compounds with elevated MRTD values generally exhibit low toxicity [44]. Furthermore, disaccharides do not pose a risk of inducing acquired long QT syndrome, as they do not inhibit hERG I and hERG II potassium channels [45]. The findings from acute and chronic oral toxicity tests indicate that a substantial quantity of these disaccharides would be required to elicit acute or long-term adverse effects [46].

None of the disaccharides demonstrated skin sensitivity or hepatotoxicity, which is a significant finding. In contrast, both ι-carrageenan and κ-carrageenan, when subjected to partial acid degradation, exhibited moderate toxicity to human hepatocytes (Fa2N-4) under culture conditions. Specifically, degraded κ-carrageenan was also found to be moderately toxic to HepG2 human hepatocytes, whereas degraded ι-carrageenan showed no toxicity [47]. This suggests that oligosaccharides derived from different carrageenans can possess hepatotoxic properties, while carrageenan disaccharides appear to exhibit minimal or no hepatotoxicity. The results observed in this study are consistent with those reported by McKim et al. [48], who demonstrated that commercial disaccharides of κ-, λ-, and ι-carrageenan (at concentrations ranging from 100 to 1000 µg/mL; Sigma-Aldrich C.O.) do not exhibit toxicity toward HepG2 cells.

The results indicate that the disaccharides did not exhibit toxicity in Minnow fish, suggesting that these organisms may possess defense mechanisms or metabolic pathways capable of handling these substances. However, the toxicity observed in more sensitive models, such as the protozoan *T. pyriformis*, highlights a different response in organisms with less complex or more vulnerable biological systems.

This discrepancy may be attributed to differences in the absorption, metabolism, or excretion of disaccharides among the studied models. In fish, enhanced bioavailability and metabolic processes may mitigate toxic effects, whereas, in protozoa, which possess simpler cellular mechanisms, disaccharides may directly disrupt vital functions, such as cell membrane integrity or essential metabolic processes.

This finding raises potential environmental concerns regarding the use of disaccharides. However, given that these are natural molecules widely distributed in nature, further in vitro and in vivo studies are necessary to clarify this possibility.

The drug similarity test is crucial for determining whether a compound possesses physicochemical properties that support oral bioavailability, thereby contributing to an acceptable pharmacokinetic profile [31,49]. Carrageenan disaccharides met most of the criteria evaluated in the proposed tests (Lipinski, Ghose, Veber, Egan, and Muegge), falling within the defined ranges, which is essential for ensuring adequate systemic exposure of the compounds [50].

Interestingly, molecular descriptors related to polarity, such as the number of hydrogen bond acceptors and the TPSA, exceeded the established limits. This high polarity may hinder the ability of these disaccharides to traverse the lipid bilayer of biological membranes and may also facilitate their recognition by P-gp [51]. Additionally, dIC, dKC, and dLC exhibited lipophilicity values (WLOGP and XLOGP3) below the established thresholds, while dLC had a molecular mass exceeding the test limit—factors that further impede permeability [52].

These results underscore the challenges disaccharides face in crossing biological membranes (including Caco-2 cells, the intestine, the blood–brain barrier, and the central nervous system), as indicated by the ADMET profile analysis. They highlight the necessity for optimizing these characteristics through structural modifications aimed at enhancing oral bioavailability and, consequently, the potential for pharmaceutical success.

The selection of molecular targets is essential for guiding the search for new drugs. In predicting the targets of disaccharides, five final targets were identified as different isoforms of carbonic anhydrases (CAs): CA I and II (located in the cytoplasm) and CA IX, XII, and XIV (located in the cell membrane). These enzymes catalyze the reversible hydration of carbon dioxide and play a critical role in regulating acid–base balance [53]. Their isoforms are present in various tissues and organs, including the lungs, kidneys, eyes, and central nervous system, and are involved in numerous physiological and pathological processes, such as gluconeogenesis, lipogenesis, ureagenesis, tumorigenicity, and the growth and virulence of several pathogens [54].

In the context of cancer, CAs, regulated by tumor hypoxia, create a differential pH environment in tumor cells, where the external pH becomes more acidic while the internal pH remains closer to normal physiological conditions. This dynamic activates a cascade of events that confer advantages for the survival and proliferation of tumor cells [55,56,57].

To promote this differential pH, Mboge and collaborators [58] propose two hypotheses. The first suggests that membrane-associated CAs (IX, XII, and XIV) work in conjunction with cytosolic CAs (CA I and II) to cycle substrates such as water, CO_2_, HCO_3_^−^, and protons. The second hypothesis posits that CAs IX and XII facilitate the dehydration of HCO_3_^−^ and the sequestration of protons, thereby acidifying the extracellular medium, while CAs I and II convert excess CO_2_ into HCO_3_^−^ to buffer intracellular pH and supply substrates for transport to the extracellular surface, where they can be utilized by CAs IX and XII.

Drugs designed to target these enzymes, such as celecoxib, imatinib, nilotinib, bortezomib, zoledronic acid, hydroxyurea, and pazopanib, inhibit their activity through various mechanisms [59,60,61,62,63,64,65]. This inhibition effectively disrupts the establishment of a tumor-friendly environment, suggesting a potential mechanism through which carrageenan disaccharides may exert their anticancer effects.

Data from docking analyses, a powerful technique for virtual screening of induced small molecules and for predicting ligand–protein interactions in computer-assisted drug development [66], reinforce this idea. In this study, it was possible to observe that the bonds made between disaccharides and CAs I, II, IX, XII, and XIV demonstrated excellent energies, since the more negative the bond, the greater the binding affinity. Furthermore, when comparing the energies of the docking made with CAs and disaccharides with those obtained in the dockings made with CAs and drugs already used for these targets, we observed that, in most cases, the docking energies with disaccharides were equal or even better, which highlights the possibility of using these disaccharides to act on these targets. In addition, interactions of these disaccharides with amino acids were identified, which, according to Mollica et al. [67], are highly conserved in these CAs. These conserved amino acids play a crucial role in the conformation of CAs and directly influence their catalytic efficiency and specificity [68,69]. The interactions of dIC and dKC with the Zn cofactor of CAs IX and II, respectively, and the interaction of dLC with the Zn of CA XII and CA XIV further support the possibility that these disaccharides effectively inhibit these CAs. This is because the interaction with the Zn cofactor is the main form of action of the inhibitors of these enzymes, since Zn is essential for their catalytic activity [70].

In molecular dynamics, a crucial approach for understanding the behavior of molecular complexes [71], the complexes dIC-CAII, dKC-CAII, dIC-CAIX, dKC-CAIX, dLC-CA XII, and dLC-CA XIV exhibited similar behaviors. The RMSD analysis, which assesses system stability, revealed that all complexes remained within a range of 0.1–0.3 nm, indicating no significant structural changes and confirming the stability of the bond between disaccharides and carbonic anhydrases [72,73,74].

Furthermore, the RMSF analysis, which predicts the individual flexibility of residues, reinforced this stability. While the terminal regions typically exhibited larger conformational variations and consequently higher RMSF values, the regions involved in ligand–protein interactions displayed smaller fluctuations, suggesting that these areas are stabilized due to molecular interactions [73,74,75].

Computer simulations are essential tools in compound screening, drug optimization, and the understanding of molecular interactions. These simulations not only help predict potential therapeutic targets but also enable the optimization of compound structures to enhance efficacy and minimize adverse effects. Despite their usefulness, computational models have inherent limitations. To address these, experimental validation is crucial, as it allows for a more comprehensive analysis of drug–molecular target interactions, ultimately accelerating the drug discovery process.

In this context, future studies aim to test the accuracy of molecular docking predictions by conducting in vitro binding assays, such as fluorescence or surface plasmon resonance (SPR), to directly assess the interaction between carrageenan disaccharides and their proposed molecular targets. Additionally, to complement molecular dynamics simulations, experiments investigating the structural stability of these interactions, such as thermal stability and enzymatic activity assays, will be conducted. Furthermore, both in vitro and in vivo experiments are planned to evaluate the therapeutic response to varying concentrations of disaccharides, providing insight into their dose–effect relationship and therapeutic window.

The data presented here suggest that carrageenan-derived disaccharides hold promise for the treatment of various types of cancer. However, significant advancements are necessary for their transition from laboratory research to clinical practice. To facilitate this transition, researchers must focus on more in-depth investigations of the pharmacokinetics and toxicity of these compounds through preclinical studies, ensuring their safety and efficacy in humans. Moreover, exploring potential synergies between carrageenan disaccharides and other anticancer therapies, such as chemotherapy or immunotherapy, could further enhance their therapeutic potential. Lastly, it is crucial that research progresses toward developing effective drug delivery systems, such as nanoparticles or controlled-release mechanisms, to ensure the targeted and efficient delivery of disaccharides to their sites of action. These steps are vital for advancing carrageenan disaccharides and oligosaccharides from experimental studies to clinical application.

## 4. Materials and Methods

### 4.1. Acquisition of the Disaccharide Structures of Iota, Kappa, and Lambda Carrageenans

The structures of iota (CID: 73155736), kappa (CID: 73155740), and lambda (CID: 91972149) carrageenans were retrieved in SMILES format from the PubChem database (https://pubchem.ncbi.nlm.nih.gov/ accessed on 15 November 2023, a comprehensive chemical information repository that consolidates data from 872 sources, with a particular focus on small molecules [76]. Subsequently, molecules that were not in disaccharide form were adjusted and reduced to their disaccharide structures using the MolView chemical compound editor [77], and saved in the same format.

### 4.2. ADMET Assessment

The PKCSM web server (https://biosig.lab.uq.edu.au/pkcsm/ accessed on 15 November 2023) was used to predict the pharmacokinetic profile of dIC, dKC, and dLC. Their structures were uploaded in SMILES format, and the properties of absorption (water solubility, Caco-2 permeability, human intestinal absorption, skin permeability, P-gp substrate or inhibitor), distribution (Vdss, BBB permeability, CNS permeability), metabolism (CYP enzymes substrate or inhibitor), excretion (total clearance and renal OCT2 substrate), and toxicity (AMES, MRTD, hERG I and II inhibitor, LD50, LOAEL, hepatotoxicity, skin sensitization, *T. pyriformis* toxicity, minnow toxicity) were assessed. This platform uses structural signatures based on graphs, which represent molecules as networks where atoms are nodes and chemical bonds are edges. These signatures capture specific structural information and chemical patterns that enable the analysis and prediction of ADMET (absorption, distribution, metabolism, excretion, and toxicity) properties [78].

### 4.3. Drug-Likeness Physicochemical Property Analysis

The drug-like properties of the disaccharides were assessed using the SwissADME server (http://www.swissadme.ch/ accessed on 15 November 2023), with their SMILES notations as input. Five commonly used drug-likeness filters were applied: Lipinski, Ghose, Veber, Egan, and Muegge, each with specific physicochemical property thresholds to evaluate drug-likeness. According to Lipinski’s rule, drug-like molecules should have a molecular weight (MW) ≤ 500 g/mol, a Moriguchi logarithm of the octanol–water partition coefficient (MLOGP) ≤ 4.15, a maximum of 5 hydrogen donors (H-donors), and up to 10 hydrogen acceptors (H-acceptors) [79]. Ghose’s filter defines drug-likeness for molecules with MW ≤ 480 g/mol, a Wildman and Crippen logarithm of the octanol–water partition coefficient (WLOGP) between −0.4 and 5.6, molar refractivity (MR) between 40 and 130, and a total atom count between 20 and 70 [80]. Veber’s criteria focus on the number of rotatable bonds (≤10) and topological polar surface area (TPSA) (≤ 140 Å^2^) [81]. The Egan filter uses two parameters, WLOGP (≤5.88) and TPSA (≤ 131.6 Å^2^), to predict oral bioavailability [82]. The Muegge filter evaluates nine properties: MW between 200 and 600 g/mol, extended logarithm of the octanol-water partition coefficient (XLOGP) between −2 and 5, TPSA ≤ 150 Å^2^, up to 7 rings, more than 4 carbon atoms, more than 1 heteroatom, up to 15 rotatable bonds, H-donors ≤ 5, and H-acceptors ≤ 10 [83]. The more criteria a compound satisfies, the higher its likelihood of being orally bioavailable [49].

### 4.4. Identification of Cancer-Related Molecular Targets

The potential molecular targets of dIC, dKC, and dLC were identified using two tools: PPB2 (https://ppb2.gdb.tools/ accessed on 15 November 2023) and SwissTargetPrediction (http://www.swisstargetprediction.ch/ accessed on 15 November 2023). PPB2 predicts targets for chemical structures by comparing their similarity to molecules registered in the ChEMBL database [84]. SwissTargetPrediction is based on the principle of chemical similarity and employs reverse screening on a library of 376,342 compounds with known experimental activity on 3068 macromolecular targets [85]. For both tools, the disaccharides were input using their SMILES notations. In PPB2, the chosen prediction method was NN(ECfp4) + NB(ECfp4). The results from both platforms were saved and cross-referenced to identify targets shared by dIC, dKC, and dLC, as well as those identified by both tools. To refine the final list of relevant targets for cancer treatment, an additional analysis was conducted to determine whether there are existing oncology drugs that act on these targets. For this, a dataset of drugs approved for cancer treatment, along with their corresponding targets, was downloaded from the Probes and Drugs database (https://www.probes-drugs.org/home/ accessed on 15 November 2023).

### 4.5. Molecular Docking

To investigate how the disaccharides (dIC, dKC, and dLC) interact with their predicted molecular targets, a molecular docking study was performed. Cancer treatment drugs approved by the NIH, known to act on some of these targets, were also included in the docking analysis, serving as references for comparative evaluation of binding energies.

Initially, the SMILES structures of the disaccharides and oncology drugs (from the dataset obtained via Probes and Drugs) were converted to PDB format using OPENBABEL [86]. Both the drugs and disaccharides were then prepared using widely recognized tools in the field, including Avogadro v1 [87] and AutoDockTools v4.2.6 [88]. These tools are commonly employed for molecular editing and optimization, facilitating the formatting of input files for docking studies. In Avogadro, hydrogen atoms were added to the structures, and the molecules were subjected to the MMFF94 force field to generate low-energy conformations. Subsequently, in AutoDockTools, Gasteiger charges were assigned, and the files were saved in PDBQT format for docking analysis.

The 3D structures of the target proteins, specifically carbonic anhydrases (CAs) I (PDB: 6i0j), II (PDB: 7rnz), IX (PDB: 6g9u), XII (PDB: 1jd0), and XIV (PDB: 5cjf), were downloaded from the Protein Data Bank (https://www.rcsb.org/) at resolutions of 1.35 Å, 1.30 Å, 1.75 Å, 1.50 Å, and 1.83 Å, respectively. The Protein Data Bank is a global repository for the deposition of large biological macromolecules, such as proteins, DNA, and RNA, essential for structural biology research [89]. The preparation of these protein structures was carried out using AutoDockTools. During this process, co-crystallized ligands were removed, polar hydrogens were added, Kollman atomic charges were assigned, and the zinc metal ion (Zn) was given a +2 charge. Finally, all target files were saved in PDBQT format, ready for molecular docking studies.

Molecular docking was carried out using AutoDock Vina v1.1.2, a widely used open-source docking program, recognized as one of the top choices in the field due to its consistent performance and improvements over time. Recent updates, particularly for carbohydrate docking (Vina-Carb), have enhanced the accuracy of its scoring function [90]. For each docking run, a grid box with dimensions of 40 × 40 × 40 Å was defined and centered at the following coordinates (x, y, z) for the respective carbonic anhydrase (CA) targets: 11.536154, 34.647962, and 16.927462 for CA I; −25.101472, 4.796194, and 13.923417 for CA II; 12.966529, −28.034500, and −54.965559 for CA IX; 18.481000, 6.524077, and 25.563923 for CA XII; and 67.757320, 37.504360, and −2.106480 for CA XIV. The docking parameters were set with an exhaustiveness of 8, a maximum of 9 models, and an energy range of 3 kcal/mol. To validate the docking methodology, redocking of the co-crystallized native ligands into their respective targets was performed. The docking results were visualized, and images were generated using the Discovery Studio v21.1.0.20298 viewer from BIOVIA.

### 4.6. Molecular Dynamics Simulations

Using GROMACS 2024.2, a widely utilized software for molecular dynamics simulations of biomolecules [91], 100 ns simulations were performed to assess the stability of the complexes where disaccharides interacted with the zinc cofactor of carbonic anhydrases: dIC-CAII, dKC-CAII, dIC-CAIX, dKC-CAIX, dLC-CAXII, and dLC-CAXIV. Protein topologies were generated using the pdb2gmx tool with the all-atom CHARMM27 forcefield [92], while ligand topologies were created using the CHARMM force field via the SwissParam server (http://swissparam.ch/) [93]. The complexes were positioned in a dodecahedral simulation box, maintaining a minimum distance of 1 nm from the box edges. The systems were solvated with the TIP3P water model [94] and neutralized by adding the appropriate counterions (Cl^−^/Na^+^). Energy minimization was conducted using the steepest descent method [95]. System equilibration was performed in two steps: first, in the canonical ensemble (NVT) for 500 ps at 300 K using a modified Berendsen thermostat, followed by the isothermal-isobaric ensemble (NPT) for 500 ps at 1 bar using a Parrinello–Rahman barostat [96]. The stability of the molecular dynamics simulations was evaluated based on RMSD and RMSF values.

### 4.7. Language Editing

For the enhancement of the English language in the manuscript, the authors utilized ChatGPT version 4.0, an AI language model developed by OpenAI (San Francisco, CA, USA). This tool was employed to improve clarity and coherence throughout the text.

## 5. Conclusions

In summary, the disaccharides dIC, dKC, and dLC demonstrated a similar pharmacological profile, presenting favorable metabolism, excretion, and toxicity for use in humans, although they face challenges related to absorption and distribution. In drug similarity tests, the disaccharides exhibited physicochemical characteristics comparable to those of approved drugs, except for the permeability of these molecules. Regarding the molecular targets associated with cancer, carbonic anhydrases (CA) I, II, IX, XII, and XIV were identified as common targets for dIC, dKC, and dLC. Molecular docking analysis revealed that the disaccharides presented binding energies comparable to or higher than those of oncology drugs targeting these targets. The complexes dIC-CAII, dKC-CAII, dIC-CAIX, dKC-CAIX, dLC-CAII, and dLC-CAIXIV stood out due to the interaction of the disaccharide with the zinc cofactor, which is the main mechanism of inhibition of these enzymes. The molecular dynamics evaluation showed that these complexes maintained a stable binding throughout the simulation. The data obtained indicate that the evaluated disaccharides have very similar pharmacological properties, making it difficult to identify a single compound with greater antitumor potential. However, this study provides relevant information on the pharmacological properties and mechanisms of action of carrageenan disaccharides in the context of cancer. It is important to emphasize that clinical trials and in vitro and in vivo experiments are necessary to validate these findings.

## Figures and Tables

**Table 1:** Analysis of the pharmacokinetic properties of iota (dIC), kappa (dKC), and lambda (dLC) carrageenan disaccharides: absorption, distribution, metabolism, and excretion (ADME).

Property	Test	Predicted Value			Parameter	Unit
		dIC	dKC	dLC		
**Absorption**	Water solubility	−2.832	−2.663	−2.892	n.a	Numeric (log mol/L)
	Caco2 permeability	−0.709	−0.611	−1.113	High if >0.90	Numeric (log Papp in 10^−6^ cm/s)
	Intestinal absorption (human)	0	6.094	0	Low if <30%	Numeric (% Absorbed)
	Skin Permeability	−2.735	−2.735	−2.735	Low if >−2.5	Numeric (log Kp)
	P-glycoprotein substrate	Yes	Yes	Yes	n.a	Categorical (Yes/No)
	P-glycoprotein I inhibitor	No	No	No	n.a	Categorical (Yes/No)
	P-glycoprotein II inhibitor	No	No	No	n.a	Categorical (Yes/No)
**Distribution**	VDss (human)	−0.647	−1.167	−0.413	Low if <−0.15 and high if >0.45	Numeric (log L/kg)
	BBB permeability	−1.873	−1.387	−2.38	Low if <−1 and high if >0.3	Numeric (log BB)
	CNS permeability	−3.69	−3.924	−3.961	Low if <−3 and high if >−2	Numeric (log PS)
**Metabolism**	CYP2D6 substrate	No	No	No	n.a	Categorical (Yes/No)
	CYP3A4 substrate	No	No	No	n.a	Categorical (Yes/No)
	CYP1A2 inhibitor	No	No	No	n.a	Categorical (Yes/No)
	CYP2C19 inhibitor	No	No	No	n.a.	Categorical (Yes/No)
	CYP2C9 inhibitor	No	No	No	n.a.	Categorical (Yes/No)
	CYP2D6 inhibitor	No	No	No	n.a.	Categorical (Yes/No)
	CYP3A4 inhibitor	No	No	No	n.a.	Categorical (Yes/No)
**Excretion**	Total Clearance	1.6	1.504	1.851	n.a.	Numeric (log mL/min/kg)
	Renal OCT2 substrate	No	No	No	n.a.	Categorical (Yes/No)

n.a.—Not applicated.

**Table 2:** Toxicity analysis of carrageenan disaccharides iota (dIC), kappa (dKC), and lambda (dLC).

Property	Test	Predicted Value			Parameter	Unit
		dIC	dKC	dLC		
**Toxicity**	AMES toxicity	No	No	No	n.a.	Categorical (Yes/No)
	Max. tolerated dose (human)	0.855	1.031	0.477	Low if ≤0.477 and high if >0.477	Numeric (log mg/kg/day)
	hERG I inhibitor	No	No	No	n.a.	Categorical (Yes/No)
	hERG II inhibitor	No	No	No	n.a.	Categorical (Yes/No)
	Oral Rat Acute Toxicity (LD50)	2.397	1.992	2.482	n.a.	Numeric (mol/kg)
	Oral Rat Chronic Toxicity (LOAEL)	3.466	3.526	4.397	n.a.	Numeric (log mg/kg_bw/day)
	Hepatotoxicity	No	No	No	n.a.	Categorical (Yes/No)
	Skin Sensitisation	No	No	No	n.a.	Categorical (Yes/No)
	*T. pyriformis* toxicity	0.285	0.285	0.285	High if >−0.5	Numeric (log ug/L)
	Minnow toxicity	8.584	6.191	14.792	High if <0.5	Numérico (log mM)

n.a.—Not applicated.

**Table 3:** Drug-likeness tests of carrageenan disaccharides iota (dIC), kappa (dKC), and lambda (dLC).

	Drug-Likeness Tests				
Molecule	Lipinski	Ghose	Veber	Egan	Muegge
**dIC**	Violation: H-acceptor > 10	Violation: WLOGP < −0.4	Violation: TPSA > 140	Violation: TPSA > 131.6	Violations: XLOGP3 < −2, TPSA > 150, H-acceptor > 10
**dKC**	Violation: H-acceptor > 10	Violation: WLOGP < −0.4	Violation: TPSA > 140	Violation: TPSA > 131.6	Violations: XLOGP3 < −2, TPSA > 150, H-acceptor > 10
**dLC**	Violations: MW > 500, H-acceptor > 10	Violations: MW > 480, WLOGP < −0.4	Violation: TPSA > 140	Violation: TPSA > 131.6	Violations: XLOGP3 < −2, TPSA > 150, H-acceptor > 10

**Table 4:** Selected targets of carrageenan disaccharides iota (dIC), kappa (dKC), and lambda (dLC).

SwissTarget			PPB2			Probes and Drugs
Target	Uniprot ID	Target Class	dIC Rank	dKC Rank	dLC Rank	Cancer Drugs with the Same Target
CA I	P00915	Lyase	3	2	4	Celecoxib, imatinib, nilotinib, bortezomib
CA II	P00918	Lyase	1	1	1	Celecoxib, imatinib, hydroxyurea, nilotinib, bortezomib, zoledronic acid
CA IX	Q16790	Lyase	2	3	2	Celecoxib, imatinib, hydroxyurea, nilotinib, pazopanib, bortezomib, zoledronic acid
CA XII	O43570	Lyase	4	4	3	Celecoxib, imatinib, nilotinib, bortezomib, zoledronic acid
CA XIV	Q9ULX7	Lyase	5	5	5	Celecoxib, imatinib, nilotinib, bortezomib, zoledronic acid

**Table 5:** Score (kcal/mol) of molecular dockings of iota carrageenan (dIC), kappa carrageenan (dKC), and lambda carrageenan (dLC) disaccharides with carbonic anhydrases (CA): CA I, CA II, CA IX, CA XII, and CA XIV.

Ligand	Receiver	Docking Score (kcal/mol)
dIC	CA I	−9.7
	CA II	−9.1
	CA IX	−8
	CA XII	−9.3
	CA XIV	−8.4
dKC	CA I	−9.2
	CA II	−8.3
	CA IX	−8.6
	CA XII	−8.8
	CA XIV	−8.5
dLC	CA I	−10.3
	CA II	−9.2
	CA IX	−8.6
	CA XII	−9.8
	CA XIV	−10.8

## Data Availability

Datasets generated during the current study are available from the corresponding author upon reasonable request.

## Supplementary Materials

The following supporting information can be downloaded at: https://www.mdpi.com/article/10.3390/md23010006/s1, Table S1: SwissTarget predicted targets for the carrageenan disaccharides iota (dCI), kappa (dCK), and lambda (dCL); Table S2: Targets predicted by PPB2 for the carrageenan disaccharides iota (dCI) kappa (dCK) and lambda (dCL); Table S3: NIH-approved drugs and their respective targets; Table S4: Scores attributed to molecular dockings performed with carbonic anhydrases (CA) I, II, IX, XII, and XIV and the oncology drugs that act on them.
