# Supplementary material for: Exploring the Pharmacological Potential of Carrageenan Disaccharides as Antitumor Agents: An In Silico Approach
Source: Mar Drugs. 2024 Dec 26;23(1):6. doi: 10.3390/md23010006 (PMC11766674; doi:10.3390/md23010006)
Supplement: Supplementary file 1 [file marinedrugs-23-00006-s001.zip › marinedrugs-3385848-supplementary S1.pdf]

Table S1: SwissTarget predicted targets for the carrageenan disaccharides iota (dCI), kappa (dCK), and lambda (dCL).

dIC				
**Target**	**Common name**	**Uniprot ID**	**Target class**	**Probability**
Acid fibroblast growth fator*	FGF1	P05230	Secreted protein	1.753.725.567
Heparanase*	HPSE	Q9Y251	Enzyme	1.488.012.776
Basic fibroblast growth fator*	FGF2	P09038	Secreted protein	1.047.189.191
Carbonic anhydrase II*	CA2	P00918	Lyase	1.047.189.191
Carbonic anhydrase I*	CA1	P00915	Lyase	1.047.189.191
Carbonic Anhydrase XII*	CA12	O43570	Lyase	1.047.189.191
Carbonic anhydrase IX*	CA9	Q16790	Lyase	1.047.189.191
Carbonic Anhydrase VII*	CA7	P43166	Lyase	1.047.189.191
Carbonic Anhydrase VI*	CA6	P23280	Lyase	1.047.189.191
Carbonic Anhydrase XIV*	CA14	Q9ULX7	Lyase	1.047.189.191
Carbonic Anhydrase IV*	CA4	P22748	Lyase	1.047.189.191
Carbonic Anhydrase XIII*	CA13	Q8N1Q1	Lyase	1.047.189.191
Carbonic Anhydrase VB*	CA5B	Q9Y2D0	Lyase	1.047.189.191
Carbonic anhydrase VA*	CA5A	P35218	Lyase	1.047.189.191
Non-secretory ribonuclease	RNASE2	P10153	Enzyme	0.0
**dKC**				
**Target**	**Common name**	**Uniprot ID**	**Target class**	**Probability**
Acid fibroblast growth fator*	FGF1	P05230	Secreted protein	0.0983232700299
Heparanase*	HPSE	Q9Y251	Enzyme	0.0788745057244
Basic fibroblast growth fator*	FGF2	P09038	Secreted protein	0.0498457217793
Carbonic anhydrase II*	CA7	P43166	Lyase	0.0498457217793
Carbonic anhydrase I*	CA6	P23280	Lyase	0.0498457217793
Carbonic Anhydrase XII*	CA14	Q9ULX7	Lyase	0.0498457217793
Carbonic anhydrase IX*	CA4	P22748	Lyase	0.0498457217793
Carbonic Anhydrase VII*	CA13	Q8N1Q1	Lyase	0.0498457217793
Carbonic Anhydrase VI*	CA5B	Q9Y2D0	Lyase	0.0498457217793
Carbonic Anhydrase XIV*	CA5A	P35218	Lyase	0.0498457217793
Carbonic Anhydrase IV*	CA2	P00918	Lyase	0.0498457217793
Carbonic Anhydrase XIII*	CA1	P00915	Lyase	0.0498457217793
Carbonic Anhydrase VB*	CA12	O43570	Lyase	0.0498457217793
Carbonic anhydrase VA*	CA9	Q16790	Lyase	0.0498457217793
Vascular endothelial growth factor A	VEGFA	P15692	Secreted protein	0.0

dLC				
**Target**	**Common name**	**Uniprot ID**	**Target class**	**Probability**
Acid fibroblast growth fator*	FGF1	P05230	Secreted protein	0.328809059523
Heparanase*	HPSE	Q9Y251	Enzyme	0.197865473018
Basic fibroblast growth fator*	FGF2	P09038	Secreted protein	0.128066504443
Carbonic anhydrase II*	CA7	P43166	Lyase	0.119347425668
Carbonic anhydrase I*	CA6	P23280	Lyase	0.119347425668
Carbonic Anhydrase XII*	CA14	Q9ULX7	Lyase	0.119347425668
Carbonic anhydrase IX*	CA4	P22748	Lyase	0.119347425668
Carbonic Anhydrase VII*	CA13	Q8N1Q1	Lyase	0.110612204199
Carbonic Anhydrase VI*	CA5B	Q9Y2D0	Lyase	0.110612204199
Carbonic Anhydrase XIV*	CA5A	P35218	Lyase	0.110612204199
Carbonic Anhydrase IV*	CA2	P00918	Lyase	0.110612204199
Carbonic Anhydrase XIII*	CA1	P00915	Lyase	0.110612204199
Carbonic Anhydrase VB*	CA12	O43570	Lyase	0.110612204199
Carbonic anhydrase VA*	CA9	Q16790	Lyase	0.110612204199
Vascular endothelial growth factor A	VEGFA	P15692	Secreted protein	0.0

* Common targets for the three disaccharides
