# Supplementary material for: Exploring the Pharmacological Potential of Carrageenan Disaccharides as Antitumor Agents: An In Silico Approach
Source: Mar Drugs. 2024 Dec 26;23(1):6. doi: 10.3390/md23010006 (PMC11766674; doi:10.3390/md23010006)
Supplement: Supplementary file 1 [file marinedrugs-23-00006-s001.zip › marinedrugs-3385848-supplementary S2.pdf]

Table S2: Targets predicted by PPB2 for the carrageenan disaccharides iota (dCI) kappa (dCK) and lambda (dCL).

**dIC**		
**Rank**	**ChEMBL ID**	**Target**
1	CHEMBL205	Carbonic Anhydrase II*
2	CHEMBL3594	Carbonic Anhydrase IX*
3	CHEMBL261	Carbonic Anhydrase I*
4	CHEMBL3242	Carbonic Anhydrase XII*
5	CHEMBL3510	Carbonic anhydrase XIV*
6	CHEMBL2804	Inositol 1,4,5-triphosphate receptor type 1*
7	CHEMBL2074	Maltase-glucoamylase*
8	CHEMBL2326	Carbonic anhydrase VII
9	CHEMBL4531	Galectin-3*
10	CHEMBL3513	Acid alpha-glucosidase*
11	CHEMBL3880	Heat shock protein HSP 90-alpha*
12	CHEMBL3114	Sucrase-isomaltase*
13	CHEMBL1973	Tyrosinase
14	CHEMBL3729	Carbonic anhydrase IV
15	CHEMBL333	Matrix metalloproteinase-2*
16	CHEMBL299	Protein kinase C alpha*
17	CHEMBL335	Protein tyrosine phosphatase 1B*
18	CHEMBL2409	Epoxide hydrolases
19	CHEMBL332	Matrix metalloproteinase-1
20	CHEMBL1900	Aldose reductase*
**dKC**		
**Rank**	**ChEMBL ID**	**Target**
1	CHEMBL205	Carbonic Anhydrase II*
2	CHEMBL261	Carbonic Anhydrase I*
3	CHEMBL3594	Carbonic Anhydrase IX*
4	cHEMBL3242	Carbonic Anhydrase XII*
5	CHEMBL3510	Carbonic anhydrase XIV*
6	CHEMBL2804	Inositol 1,4,5-triphosphate receptor type 1*
7	CHEMBL2326	Carbonic anhydrase VII
8	CHEMBL3513	Acid alpha-glucosidase*
9	CHEMBL4531	Galectin-3*
10	CHEMBL3114	Sucrase-isomaltase*
11	CHEMBL1973	Tyrosinase
12	CHEMBL2074	Maltase-glucoamylase*
13	CHEMBL3729	Carbonic anhydrase IV
14	CHEMBL333	Matrix metalloproteinase-2*
15	CHEMBL335	Protein tyrosine phosphatase 1B*

16	CHEMBL3880	Heat shock protein HSP 90-alpha*
17	CHEMBL299	Protein kinase C alpha*
18	CHEMBL332	Matrix metalloproteinase-1
19	CHEMBL1900	Aldose reductase*
20	CHEMBL2608	Lysosomal alpha-glucosidase
**dLC**		
**Rank**	**ChEMBL ID**	**Target**
1	CHEMBL205	Carbonic Anhydrase II*
2	CHEMBL3594	Carbonic Anhydrase IX*
3	CHEMBL3242	Carbonic Anhydrase XII *
4	CHEMBL261	Carbonic Anhydrase I*
5	CHEMBL3510	Carbonic Anhydrase XIV*
6	CHEMBL4531	Galectin-3*
7	CHEMBL299	Protein kinase C alpha*
8	CHEMBL3513	Acid alpha-glucosidase*
9	CHEMBL2804	Inositol 1,4,5-triphosphate receptor type 1*
10	CHEMBL3114	Sucrase-isomaltase*
11	CHEMBL5474	Galectin-9
12	CHEMBL1770047	Low-affinity sodium-glucose cotransporter
13	CHEMBL2074	Maltase-glucoamylase*
14	CHEMBL1900	Aldose reductase*
15	CHEMBL4518	P2Y14 purinergic receptor
16	CHEMBL333	Matrix metalloproteinase-2*
17	CHEMBL335	Protein tyrosine phosphatase 1B*
18	CHEMBL3880	Heat shock protein HSP 90-alpha*
19	CHEMBL5008	Galectin-7
20	CHEMBL2622	Aldose reductase (rat)

* Common targets for the three disaccharides
