# Supplementary material for: Exploring the Pharmacological Potential of Carrageenan Disaccharides as Antitumor Agents: An In Silico Approach
Source: Mar Drugs. 2024 Dec 26;23(1):6. doi: 10.3390/md23010006 (PMC11766674; doi:10.3390/md23010006)
Supplement: Supplementary file 1 [file marinedrugs-23-00006-s001.zip › marinedrugs-3385848-supplementary S4.pdf]

Table S4: Scores attributed to molecular dockings performed with carbonic anhydrases (CA) I, II, IX, XII and XIV and the oncology drugs that act on them.

Targets	Cancer drugs for this target	Docking score (kcal/mol)
CA I	Celecoxib	-8.6
	Imatinib	-9.1
	Nilotinib	-9.6
	Bortezomib	-8.7
CA II	Celecoxib	-9.6
	Imatinib	-9.5
	Hydroxyurea	-4.3
	Nilotinib	-11.4
	Bortezomib	-8.8
	Zoledronic acid	-8.1
CA IX	Celecoxib	-9.4
	Imatinib	-9.5
	Hydroxyurea	-4.4
	Nilotinib	-9.3
	Pazopanib	-9.1
	Bortezomib	-8.3
	Zoledronic acid	-6.3
CA XII	Celecoxibe	-9.0
	Imatinibe	-8.7
	Nilotinibe	-10.0
	Bortezomibe	-8.7
	Ácido zoledrônico	-6.7
CA XIV	Celecoxib	-9.9
	Imatinib	-9.2
	Nilotinib	-10.6
	Bortezomib	-9.3
	Zoledronic acid	-6.7
